# Operant behavior is reliably impaired in a mouse model of Angelman syndrome

**DOI:** 10.21203/rs.3.rs-9043922/v1

**Published:** 2026-03-20

**Authors:** Caleigh D. Guoynes, Devante D. Kerr, Zahraa S. Hotait, Joseph K. Tanas, Michael S. Sidorov

**Affiliations:** 1 Center for Neuroscience Research, Children’s National Hospital, Washington, DC, USA; 2 Departments of Pediatrics and Pharmacology & Physiology, The George Washington University School of Medicine and Health Sciences, Washington, DC, USA

**Keywords:** Angelman syndrome, mouse behavior, neurodevelopmental disorders, Ube3a

## Abstract

Angelman syndrome (AS) is a neurodevelopmental disorder characterized by cognitive impairment, absent speech, seizures, motor and sleep impairments, and caused by loss of function of the maternal copy of the *UBE3A* gene. Modeling the AS phenotype in preclinical models across behavioral domains is critical for testing of new therapeutics. Our previous work reported behavioral phenotypes in male *Ube3a*^*m*−/*p*+^ (AS model) mice on a cognitive-like operant extinction task. Here, we evaluated operant task performance in male and female *Ube3a*^*m*−/*p*+^ mice across two cohorts, and correlated operant performance with performance on a battery of other widely used behavioral tests for *Ube3a* mutants. *Ube3a*^*m*−/*p*+^ mice showed impairments in both the acquisition and extinction phases of operant testing that were not sex-specific. Inclusion of operant testing in the existing *Ube3a*^*m*−/*p*+^ behavioral battery was feasible and improved clustering of genotypes in principal component space. Overall, operant acquisition and extinction testing is a reliable approach to quantify cognitive-like learning impairments in *Ube3a*^*m*−/*p*+^ mice. Operant testing should be considered as part of a broad toolbox for evaluating the effectiveness of AS treatments preclinically.

## INTRODUCTION

Angelman syndrome (AS) is a single-gene neurodevelopmental disorder marked by motor and cognitive impairment, developmental delay, absence of speech, seizures, sleep disturbances, and differences in social affective behavior^1,2^. Cognitive impairment in individuals with AS is pervasive, affecting quality of life for affected individuals and caregivers^3–7^. AS is caused by a loss of functional maternal *UBE3A* expression due to deletion, mutation, imprinting center defect, or paternal uniparental disomy^8,9^. *UBE3A* is paternally imprinted in neurons^10–12^, and approaches to re-express *UBE3A* via unsilencing the dormant paternal allele are now under evaluation in clinical trials^13–15^. As ongoing work identifies additional mechanism-based approaches to restore *UBE3A* function^16–28^ and/or target downstream pathways^29–38^, preclinical testing of potential therapies remains critical.

Behavioral testing in *Ube3a*^*m*−/*p*+^ mice^39^ is a widely used approach to evaluate the preclinical effectiveness of potential AS therapies. This mouse model has strong construct validity, as *Ube3a* imprinting is conserved in mice^11^, and it has also shown good face validity across a number of behavioral domains^40^. For example, *Ube3a*^*m*−/*p*+^ mice show impairments in motor function^39,41–44^, increased seizure susceptibility^45–47^, abnormal sleep and circadian rhythms^48–51^, and abnormal EEGs^52–55^ that are generally consistent with patient populations. In addition, *Ube3a*^*m*−/*p*+^ mice show impaired vocalizations in multiple behavioral contexts that are consistent with impaired speech in AS^56,57^. Recently, many groups have used a “gold standard” behavioral testing battery^58^ to evaluate the effectiveness of multiple types of interventions^21,25,28,38,59–62^. The main advantage of this battery, which typically includes weight, rotarod, open field, marble burying, nest building, and forced swim, is that behavioral phenotypes are robust and consistent across cohorts of mice and different labs. However, a limit of using this battery alone is its overreliance on motor or motor-adjacent behaviors. More broadly, preclinical testing in AS has been limited by challenges in reliably evaluating cognitive or cognitive-like tests. Modeling cognitive impairment is inherently challenging in mice, and thus, new *Ube3a*^*m*−/*p*+^ rat and pig models have been developed that are capable of more complex behavior^63–65^. However, evaluating cognitive-like behavior in mice remains important, as there are more advanced genetic tools in mice and many preclinical studies first screen drugs in mice.

Modeling cognitive impairment in *Ube3a*^*m*−/*p*+^ mice can be approached in several ways. One approach is to use cognitive-like learning assays, such as novel object recognition, contextual fear conditioning, and the Morris water maze. *Ube3a*^*m*−/*p*+^ mice generally show impairments on these tests^44,66–71^, though phenotypes are typically less reliable than the gold standard battery. However, these tasks are primarily dependent on the hippocampus and other subcortical brain structures^72–74^, which limits their interpretability as a broader assessment of cognitive impairment. An alternative approach for modeling cognitive impairment is to use operant learning tasks, such as operant acquisition and extinction and the five-choice serial reaction time test, that actively engage prefrontal circuits^75–77^. The prefrontal cortex (PFC) is central to cognition and critical for high-level tasks in both humans and mice^78,79^, and *Ube3a*^*m*−/*p*+^ mice show both electrophysiological impairments in PFC and behavioral impairments on operant tasks linked to PFC function^80–83^. Our prior work reported exaggerated extinction and a trend towards delayed acquisition of an operant learning task in adult male *Ube3a*^*m*−/*p*+^ mice^83^. Here, we sought to evaluate the reliability and suitability of operant behavior for preclinical testing by assessing performance in male and female mice from multiple behavioral cohorts.

In addition, a secondary goal of this study was to evaluate the feasibility and value of adding operant testing to the existing *Ube3a*^*m*−/*p*+^ behavioral battery. We previously demonstrated that using principal component analysis, performance on the gold standard *Ube3a*^*m*−/*p*+^ behavioral battery can be summarized as a single “Angelman severity score” (PC1) that is sensitive to treatment^84^. Here, we performed operant testing and the behavioral battery concurrently, and asked whether inclusion of operant measures in multidimensional analysis improved our ability to distinguish *Ube3a* genotype based on behavior. This approach is conceptually and methodologically similar to our recent finding that including ultrasonic vocalization testing improves the breadth and effectiveness of the *Ube3a*^*m*−/*p*+^ behavioral battery^57^.

## RESULTS

### Operant acquisition and extinction are abnormal in Ube3a^m−/p+^ mice

**Figure 1.**
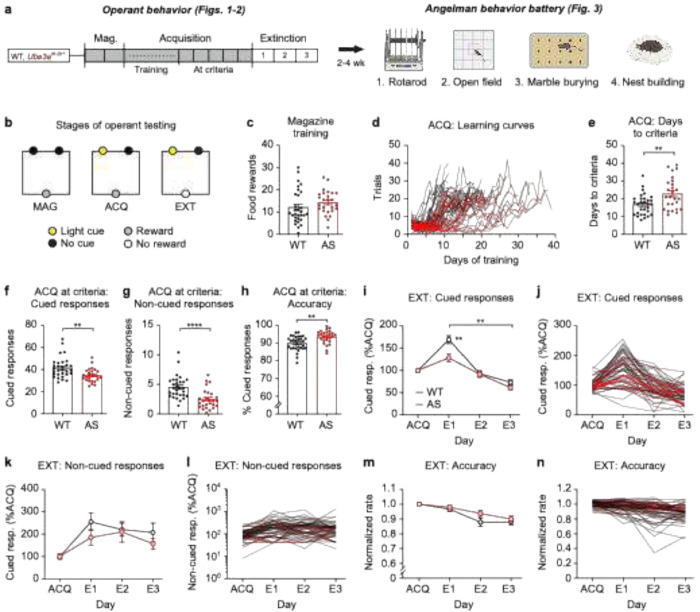
Operant acquisition and extinction phenotypes in *Ube3a*^*m*−/*p*+^ mice. (a) Experimental timeline for evaluating operant behavior and the Angelman behavior battery in the same animals. (b) Schematic of three stages of operant testing: magazine training (MAG), acquisition (ACQ), extinction (EXT). (c) No difference in magazine training between WT and *Ube3a*^*m*−/*p*+^ mice. (d) Learning curves during acquisition. (e) *Ube3a*^*m*−/*p*+^ mice took more days to reach acquisition criteria. (f) *Ube3a*^*m*−/*p*+^ mice had fewer cued responses at criteria. (g) *Ube3a*^*m*−/*p*+^ mice had fewer non-cued responses at criteria. (h) *Ube3a*^*m*−/*p*+^ mice were more accurate at criteria. (i) *Ube3a*^*m*−/*p*+^ mice made fewer cued responses on extinction day 1. Bracket indicates day × genotype interaction; E1 asterisks represent *post hoc* test. (j) Cued responses during extinction in individual mice. (k) No genotype difference in non-cued responses during extinction. (l) Non-cued responses during extinction in individual mice. Four instances of zero non-cued responses are not plotted here because data are shown on a log scale, but were included in group analysis in K. (m) No genotype difference in accuracy during extinction. (n) Accuracy during extinction in individual mice. Data represent mean ± SEM; ****p < 0.0001; **p < 0.01; black: WT (*n* = 30 mice), red: *Ube3a*^*m*−/*p*+^ (AS; *n* = 26).

We performed operant behavioral testing followed by a well-established behavior battery in adult male and female *Ube3a*^*m*−/*p*+^ mice and littermate controls (Fig. 1a). Operant testing had three distinct stages (Fig. 1b): (1) magazine training, where food-deprived mice learned that pellet rewards were delivered via the food magazine; (2) acquisition, where a nose-poke into the light-cued aperture triggered reward delivery via the magazine; (3) extinction, where nose-pokes no longer triggered reward delivery. First, we analyzed the entire dataset together, which included two cohorts of mice, and males and females in equal ratios. There was no genotype difference in the number of pellets received during magazine training (Fig. 1c; t_(54)_ = 1.240, *p* = 0.2202), suggesting no gross difference in motivation. During acquisition, mice learned across days (Fig. 1d) and advanced to extinction after performing at criteria (>15 trials, >50% accuracy) for five consecutive days. *Ube3a*^*m*−/*p*+^ mice took longer to reach operant acquisition learning criteria (Fig. 1e; t_(54)_ = 3.122, *p* = 0.0030). At criteria, *Ube3a* mice had fewer cued responses (Fig. 1f; t_(54)_ = 3.245, *p* = 0.0020) and fewer non-cued responses (Fig. 1g; t_(54)_ = 4.240, *p* < 0.0001), resulting in a higher accuracy (Fig. 1h; t_(54)_ = 3.380, *p* = 0.0014). Because response rates during acquisition were different by genotype, extinction was normalized to acquisition as in prior work^83^.

Extinction of cued responses (Fig. 1i–j; main effect of time: F_(1.965,106.1)_ = 92.90, *p* < 0.0001) was exaggerated in *Ube3a*^*m*−/*p*+^ mice (**Fig. i-j**; main effect of genotype: F_(1,54)_ = 6.078, *p* = 0.0169; genotype × time interaction: F_(2,108)_ = 5.621, *p* = 0.0048). *Post hoc* tests revealed a significant group difference on extinction day 1 (*p* = 0.0048). Non-cued responses during the extinction phase showed no genotype differences (Fig. 1k–l; main effect of genotype: F_(1, 54)_ = 1.371, *p* = 0.2469; genotype × time interaction: F_(2, 108)_ = 0.4535, *p* = 0.6366). Accuracy during extinction also showed no genotype differences (main effect of genotype: F_(1, 54)_ = 1.893, *p* = 0.1745; genotype × time interaction: F_(2, 108)_ = 1.187, *p* = 0.3090) (Fig. 1m–n). Extinction is plotted using raw data in Supplemental Figure 1. Overall, these results are consistent with our previous finding that *Ube3a*^*m*−/*p*+^ mice are more accurate at criteria during operant acquisition and that operant extinction is enhanced in *Ube3a*^*m*−/*p*+^ mice. However, in this cohort, we also found that *Ube3a*^*m*−/*p*+^ mice took longer to reach acquisition criteria.

### Operant behavioral phenotypes in Ube3a^m−/p+^ mice are generally consistent across experimental cohorts

**Figure 2.**
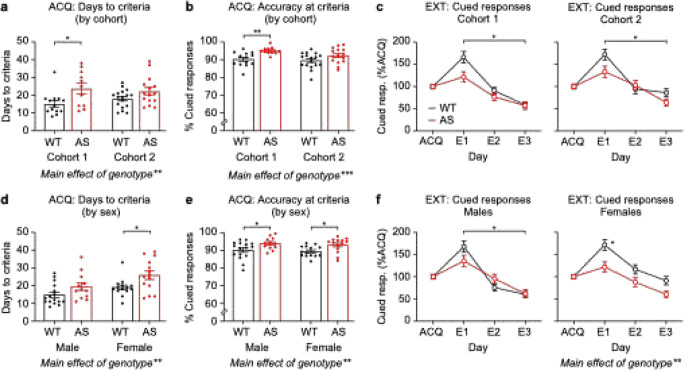
Evaluation of operant phenotypes in *Ube3a*^*m*−/*p*+^ mice by cohort and sex. (a) Acquisition days to criteria and (b) accuracy at criteria by cohort. (c) Left: cued responses during extinction in Cohort 1. Right: cued responses during extinction in Cohort 2. (d) Days to criteria and (e) accuracy at criteria by sex. (f) Left: cued responses during extinction in males. Right: cued responses during extinction in females. Data represent mean ± SEM; **p < 0.01; *p < 0.05; black: WT, red: *Ube3a*^*m*−/*p*+^ (AS). Brackets in C and F represent day × genotype interaction, other asterisks represent *post hoc* tests. For panels a-c, [Cohort 1: WT (*n* = 13 mice), AS (*n* = 11 mice); Cohort 2: WT (*n* = 17 mice), AS (*n* = 15 mice)]. For panels d-f, [Males: WT (*n* = 16 mice), AS (*n* = 12 mice); Females: WT (*n* = 14 mice), AS (*n* = 14 mice)]. When there is a main effect of genotype, it is indicated with text under the graph.

Next, we asked whether operant *Ube3a*^*m*−/*p*+^ behavioral phenotypes (days to criteria, accuracy at criteria, extinction) were consistent across experimental cohorts and sex. We compared behavior in Cohort 1 (WT: *n* = 13, *Ube3a*^*m*−/*p*+^: *n* = 11) and Cohort 2 (WT: *n* = 17, *Ube3a*^*m*−/*p*+^: *n* = 15), which were run using the same equipment by two separate experimenters. Cohort 2 used slightly smaller pellets for reward delivery (14 mg) than Cohort 1 (20 mg). For operant acquisition (days to criteria), there was a significant main effect of genotype (Fig. 2a; F_(1, 52)_ = 10.38, *p* = 0.0022) and no main effect of cohort (F_(1, 52)_ = 0.1209, *p* = 0.7295) or cohort × genotype interaction (F_(1, 52)_ =1.168, *p* = 0.2848). *Post hoc* tests revealed a statistically significant effect in Cohort 1 (*p* = 0.0127) but not Cohort 2 (*p* = 0.2037). For accuracy at acquisition criteria, there was a significant main effect of genotype (Fig. 2b; F_(1, 52)_ = 12.48, *p* = 0.0009) and no main effect of cohort (F_(1, 52)_ = 2.654, *p* = 0.1094) or cohort × genotype interaction (F_(1, 52)_ =0.8380, *p* = 0. 3642). *Post hoc* tests revealed a statistically significant effect in Cohort 1 (*p* = 0.0098) but not Cohort 2 (*p* = 0.0988). For extinction of cued responses, there was a significant day × genotype interaction in both Cohort 1 (Fig. 2c; main effect of genotype: F_(1, 22)_ = 3.873, *p* = 0.0618; interaction: F_(2, 44)_ = 3.960, *p* = 0.0262; *post hoc*: E1 *p* = 0.0575, E2 *p* = 0.5218, E3 *p* = 0.9979) and Cohort 2 (Fig. 2c; main effect of genotype: F_(1, 30)_ = 2.603, *p* = 0.1171; interaction: F_(2, 60)_ = 3.554, *p* = 0.0348; *post hoc*: E1 *p* = 0.0962, E2 *p* = 0.9226, E3 *p* = 0.2119). Across cohorts, the cued extinction response was the most reliable. Although the days to criteria and accuracy at criteria during acquisition both had main effects of genotype, *post hoc* tests were only significant for Cohort 1.

### Operant behavioral phenotypes are generally consistent in Ube3a^m−/p+^ males and females

We then compared behavior in males (WT: *n* = 16, *Ube3a*^*m*−/*p*^: *n* = 12) and females (WT: *n* = 14, *Ube3a*^*m*−/*p*^: *n* = 14) across the full dataset. For operant acquisition (days to criteria), there was a significant main effect of genotype (Fig. 2d; F_(1, 52)_ = 9.506, *p* = 0.0033) and a main effect of sex (F_(1,52)_ = 8.174, *p* = 0.0061), with females taking longer to learn the task, but no genotype × sex interaction (F_(1, 52)_ =0.3673, *p* = 0.5471).

*Post hoc* tests revealed a statistically significant effect in females (*p* = 0.0227) but not males (*p* = 0.1670). For accuracy at acquisition criteria, there was a significant main effect of genotype (Fig. 2e; F_(1,52)_ = 11.41, *p* = 0.0014) and no main effect of sex (F_(1,52)_ = 0.5311, *p* = 0.4694) or genotype × sex interaction (F_(1,52)_ = 0.00076, *p* = 0.9780). *Post hoc* tests revealed statistically significant genotype differences in both males (*p* = 0.0401) and females (*p* = 0.0415). For extinction of cued responses, there was a significant day × genotype interaction in males (Fig. 2f; main effect of genotype: F_(1, 26)_ = 0.1342, *p* = 0.7171; interaction: F_(2, 52)_ =4.422, *p* = 0.0168; *post hoc*: E1 *p* = 0.2167, E2 *p* = 0.3421, E3 *p* = 0.9985). Females showed a significant main effect of genotype (Fig. 2F; F_(1, 26)_ = 9.712, *p* = 0.0044; *post hoc*: E1 *p* = 0.0205, E2 *p* = 0.1705, E3 *p* = 0.0560) but no day × genotype interaction (F_(2, 52)_ = 1.103, *p* = 0.3395). Overall, the operant acquisition and extinction phenotypes were robust in both males and females; however, the increased days to acquisition were only significant for females.

### Operant behavior complements and enhances the existing Ube3a^m−/p+^ behavioral battery

**Figure 3.**
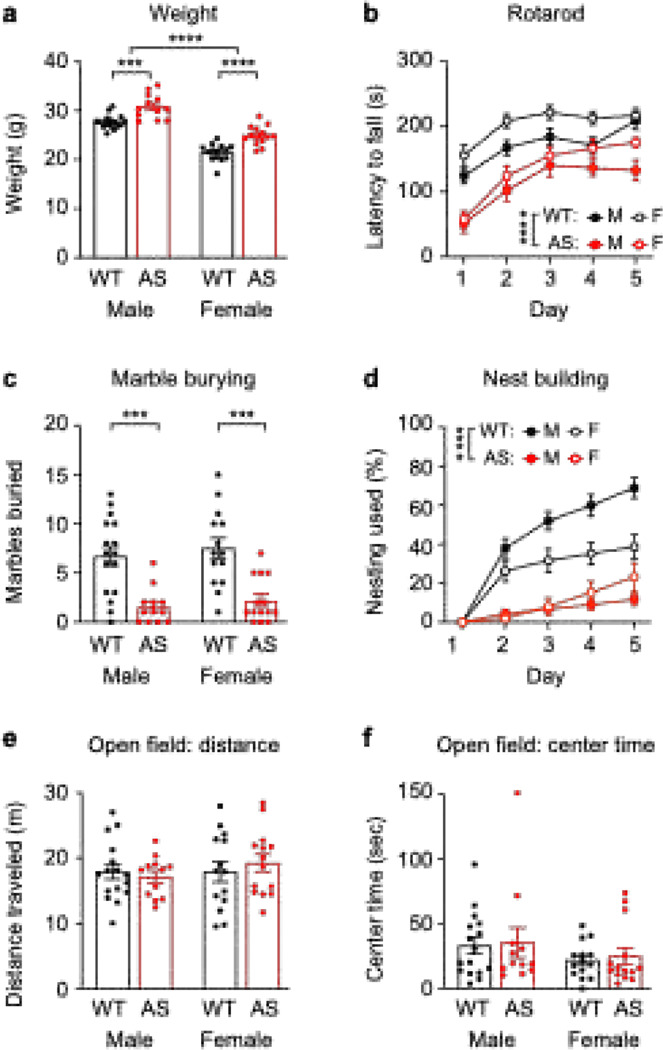
*Ube3a*^*m*−/*p*+^ mice show behavioral impairments on a widely used behavioral battery. (a) *Ube3a*^*m*−/*p*+^ mice were heavier than WT littermates. Top bracket indicates main effect of sex; other asterisks represent *post hoc* tests. (b) *Ube3a*^*m*−/*p*+^ mice had a decreased latency to fall in rotarod performance across days. Asterisks represent main effect of genotype. (c) *Ube3a*^*m*−/*p*+^ mice buried fewer marbles during the marble burying task. Asterisks indicate *post hoc* tests. (d) *Ube3a*^*m*−/*p*+^ mice used less nesting material across days. Asterisks indicate main effect of genotype. (e) No difference in open field distance between genotypes. (e) No difference in open field center time between genotypes. Data represent mean ± SEM; ****p < 0.0001; ***p < 0.001; **p < 0.01; *p < 0.05; black: WT, red: *Ube3a*^*m*−/*p*+^ (AS).

Next, we sought to evaluate the added value of including operant behavioral testing into the existing motor-focused *Ube3a*^m−/p+^ behavioral battery. To do this, we performed the battery (weight, rotarod, open field, marble burying, next building) in the same animals after operant testing concluded. We then correlated operant measures with overall performance on the *Ube3a*^*m*−/*p*+^ behavioral battery. As expected, *Ube3a*^*m*−/*p*+^ mice showed impaired performance on individual tests in this battery. *Ube3a*^*m*−/*p*+^ mice were heavier (Fig. 3a; main effect of genotype: F_(1, 52)_ = 40.69, *p* < 0.0001), showed impaired performance on the rotarod (Fig. 3b; main effect of genotype: F_(1, 52)_ = 42.33, *p* < 0.0001), marble burying (Fig. 3c; main effect of genotype: F_(1, 52)_ = 35.47, *p* < 0.0001), and nest building (Fig. 3d; main effect of genotype: F_(1, 52)_ = 54.50, *p* < 0.0001) tests. Surprisingly, *Ube3a*^*m*−/*p*+^ mice did not show a phenotype in open field testing (Fig. 3e; main effect of genotype: F_(1, 52)_ = 0.6670, *p* = 0.4178, Fig. 3f; main effect of genotype: F_(1, 52)_ = 0.1391, *p* = 0.7107). All statistical testing from Figure 3 is reported in Supplementary File 1. These results are generally consistent with prior findings from our group and other groups^58,84^, except that mice that had previously performed operant testing did not show a genotype difference in distance traveled in subsequent open field testing.

**Figure 4.**
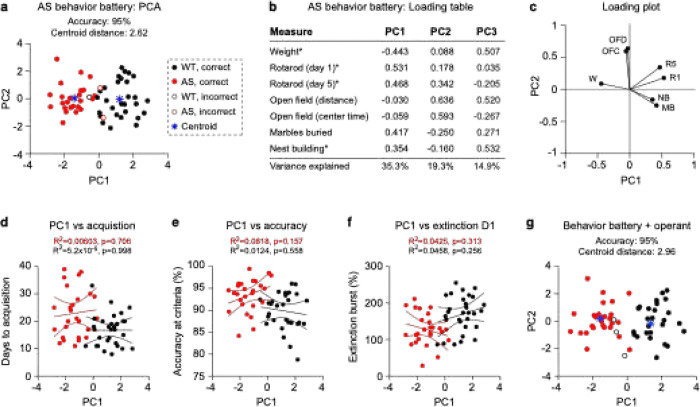
Operant behaviors are independent of AS battery behaviors and modestly enhance genotype discrimination. (a) Multidimensional analysis using data from the *Ube3a*^*m*−/*p*+^ behavior battery demonstrates accurate clustering of animals using two principal components. (b) Loading table and (c) loading plot for PCA in panel a. (d) Days to acquisition does not correlate with PC1. (e) Accuracy at criteria does not correlate with PC1. (f) The extinction burst response on day 1 does not correlate with PC1. Lines show linear fits with 95% confidence intervals. (g) Adding operant extinction behaviors to PCA analysis increases centroid distance between WT and *Ube3a*^*m*−/*p*+^ clusters. Centroids and centroid distance are calculated based on actual genotypes, not k-means clusters.

We next performed principal component analysis on the standard battery and assessed correlations with operant performance. Multidimensional analysis of standard behavioral battery assessments (weight, rotarod day 1, rotarod day 5, open field distance, open field center time, marble burying, and nest building) predicted *Ube3a* genotype with 95% accuracy (Fig. 4a; 53/56 mice). The distance between centroids representing each genotype was 2.62 units in 2PC space. All behavioral assessments with significant group differences loaded strongly onto PC1 (Fig. 4b–c), confirming our prior finding that PC1 can be used a composite Angelman behavioral severity score in mice^84^. We next asked whether PC1 correlates with each operant behavior phenotype (days to acquisition, accuracy at acquisition, day 1 extinction performance) within animals. No operant measures were significantly correlated with PC1. Days to acquisition showed no relationship with PC1 in either AS ( R^2^= 0.0003, *p* = 0.798) or WT (R^2^ = 5.2×10^−6^, *p* = 0.998) mice (Fig. 4d). Similarly, accuracy at criteria was not correlated with PC1 in either AS (R^2^ = 0.0818, *p* = 0.157) or WT (R^2^ = 0.0124, *p* = 0.558) mice (Fig. 4e). Extinction day 1 performance also showed no significant correlation with PC1 in either AS (R^2^ = 0.0425, *p* = 0.313) or WT (R^2^ = 0.0458, *p* = 0.256) mice (Fig. 4f). These findings suggest that operant testing captures behavioral variance that is unique from the standard battery. To test this hypothesis, we asked whether including operant assessments in multidimensional analysis would improve its effectiveness. Improved effectiveness could be evaluated either by an increased accuracy or by WT and AS clusters moving farther apart in PC space. Inclusion of operant measures in multidimensional analysis resulted in a consistent high accuracy (95%), while increasing the centroid distance between WT and AS clusters from 2.62 to 2.96 units in PC space (Fig. 4g). These results suggest that operant extinction measures provide complementary information to the existing behavioral battery and could enhance the sensitivity of multidimensional phenotyping approaches for detecting treatment effects in *Ube3a*^*m*−/*p*+^ mice.

### Sample size guidance for future operant studies

To guide the design of future operant studies in *Ube3a*^*m*−/*p*+^ mice, we performed power analyses to determine the sample size required to detect statistically significant effects (*α* = 0.05) for different levels of power (1-*β* = 0.8, 0.9, and 0.95) on operant behavioral measures. For each sample size (n = 4–30), we ran 10,000 iterations of a bootstrap analysis to determine the minimum number of mice per group. At 80% power, sample sizes ranged from n = 20–24 per measure in operant behavior, and from n = 7–12 for measures in the standard behavior battery (excluding open field, as we did not observe a phenotype). At 90% power, sample sizes increased to n = 26–30 per measure for operant behavior and n = 9–15 for measures in the standard behavior battery. At 95% power, sample sizes were greater than 30 per group for operant behaviors but ranged from n = 11–18 for measures in the standard battery (Table 1).

## DISCUSSION

Overall, our results confirm that operant learning is impaired in *Ube3a*^*m*−/*p*+^ mice and demonstrate that operant phenotypes are not sex-dependent. Relative to WT littermates, *Ube3a*^*m*−/*p*+^ mice took longer to learn the operant acquisition task (Fig. 1e), had higher accuracy at criteria (Fig. 1h), and showed an exaggerated extinction of cued responses on extinction day 1 (Fig. 1i). Together, these phenotypes suggest impaired cognitive function during operant learning. Within animals, operant performance did not correlate with overall performance on the gold-standard behavior battery (Figs. 4d–f), suggesting that operant testing may capture a different behavioral domain from existing motor-like tasks in *Ube3a*^*m*−/*p*+^ mice. Consistent with this idea, including operant measures increased the centroid distance between WT and *Ube3a*^*m*−/*p*+^ mice in principal component space (Fig. 4g), suggesting that these tasks further distinguished the *Ube3a*^*m*−/*p*+^ phenotype. These data demonstrate that, in addition to being a reliable test, operant extinction captures distinct cognitive-like impairments in *Ube3a*^*m*−/*p*+^ mice.

This study builds on recent operant behavioral findings in *Ube3a*^*m*−/*p*+^ mice. We replicated our previous findings that *Ube3a*^*m*−/*p*+^ male mice have enhanced operant extinction and greater accuracy at criteria^83^, and showed that with sufficient sample sizes, we can also detect significant differences in days to acquisition. While previous studies used only male mice, our current study included both male and female mice. We found that no operant phenotypes were sex dependent, which was valuable to rule out because previous studies in *Ube3a*^*m*−/*p*+^ mice found sex differences in complex behaviors^85,86^. Accuracy at criteria and extinction phenotypes were present independently in males and females, but days to acquisition was statistically significant only in female *Ube3a*^*m*−/*p*+^ mice, suggesting that the effect size may be greater in females. Similarly, when we broke operant phenotypes down by each of our two cohorts, not all phenotypes were present individually in each cohort. Only exaggerated extinction on day 1 was independently significant in each cohort (Fig. 2c). Acquisition accuracy and days to criteria were independently significant in cohort 1, but not cohort 2 (Figs. 2a–b). Breaking down our results by cohort and sex revealed that, to detect significant effects, much larger sample sizes (n=20+) are needed than for behaviors in the gold-standard behavior battery (Table 1).

In practice, there are several advantages and disadvantages to operant testing when considering this approach for preclinical testing in *Ube3a* mutants. A major advantage of operant testing is the ability to reliably test cognitive-like behavior. The commonly used behavior battery for *Ube3a*^*m*−/*p*+^ mice^58^ emphasizes motor tasks, and our results suggest that operant testing captures a different aspect of the AS phenotype. We demonstrate that operant testing can feasibly precede the gold-standard behavior battery and that adding a cognitive-like task to this battery can help distinguish the *Ube3a*^*m*−/*p*+^ phenotype in principal component space. However, operant testing has several key limitations that should guide usage. First, the long test duration (often 4+ weeks of daily testing) provides a practical barrier. While operant learning/extinction is less time-consuming than other prefrontally encoded tasks, such as the five-choice serial reaction time test (~3 months)^58^, it is more time-consuming than hippocampally encoded cognitive-like tasks, such as novel object recognition (~4 days)^87^. Another disadvantage is the large sample size required to detect effects. We report that the sample size needed to find a statistically significant effect (*α* = 0.05) and (1-*β* = 0.8) was n = 24 mice for days to acquisition, n = 19 for accuracy at criteria, and n = 21 for extinction. Thus, our two cohorts combined have sufficient power to detect an effect (WT: n = 30; *Ube3a*^*m*−/*p*+^: n = 26), but each cohort individually was underpowered (Cohort 1: WT: n = 13; *Ube3a*^*m*−/*p*+^: n = 11; WT: n = 17; *Ube3a*^*m*−/*p*+^: n = 15). The sample size needed to detect a significant effect is much higher than that required for many of the tests in the gold-standard behavior battery, making it more costly and time-consuming to run. Overall, it may be best practice to run the gold-standard behavior battery first and in a separate cohort, and then only run operant behavior if cognitive performance is an important secondary measure.

Limited face validity is another major limitation of operant extinction testing in *Ube3a*^*m*−/*p*+^ mice. While impairments in task acquisition can be easily interpreted, it is less clear how greater accuracy at task acquisition and enhanced extinction in mice translate directly to cognitive dysfunction in people with AS. An operant behavioral task in mice with potentially greater translatability is the five-choice serial reaction time test (5CSRTT). We previously demonstrated that *Ube3a*^*m*−/*p*+^ mice had increased omissions during 5CSRTT training, suggesting that attentional behavior is impaired^82^. However, 5CSRTT is much longer and more labor-intensive than operant extinction, and is more susceptible to the confounds of motor dysfunction^82^. The trade-offs between face validity and task complexity and duration remain a challenge for studying cognition in mice. While operant behavioral testing in mice has limited face validity in patient populations, it does provide a useful readout of prefrontal circuit function^82,83,88–90^ and a reliable assessment of cognitive-like performance.

A surprising finding from this study was that *Ube3a*^*m*−/*p*+^ mice did not show hypoactivity during open field testing (Fig. 3e). Hypoactivity in the open field is typically one of the more reliable phenotypes in *Ube3a* mutants^54,58,86,91^, and our group has previously reported open field hypoactivity using the same arena ^84^and protocol. We hypothesize that the lack of an open field phenotype could be driven by weeks of prior experience in a similar environment – the operant chamber – during prior operant testing. Both the operant testing box and the open field testing box are large, soundproof chambers with square behavior arenas. Prior work has demonstrated that total distance traveled in the open field decreases with experience^92,93^. We hypothesize that experimental mice habituated to the operant testing environment, resulting in decreased distance traveled in a similar open field arena. Indeed, the lack of open field phenotype was driven mainly by decreased distance traveled in WT mice (~20 meters per session in this study vs. ~40 meters in prior work)^84^. This finding has practical implications for running the behavior battery and suggests that best practice may be to run the battery separately from operant extinction if the open field phenotype is important to measure.

Overall, we demonstrate that operant behavioral phenotypes are consistent in *Ube3a*^*m*−/*p*+^ males and females, and include both differences in acquisition and extinction. Operant testing is feasible in conjunction with other behavioral tests, but it may be best to run operant extinction and the behavior battery in separate cohorts. Operant behavior may be a strong readout for measuring cognitive performance and prefrontal dysfunction, and could be a valuable tool for mechanistic studies and screening therapeutic drugs in AS. This test is likely not suitable to be added to every behavior battery because it is labor-intensive and does not have strong face validity, unlike other behavioral tests such as ultrasonic vocalizations^91^ that are also reliable and add an additional behavioral domain but are much easier to implement and have stronger translatability.

## METHODS

### Animals

All experimental protocols were approved by the Institutional Animal Care and Use Committee (IACUC) of Children’s National Hospital, and were performed in accordance with all relevant guidelines and regulations. All experiments in this study complied with the ARRIVE guidelines^94^. All datasets used *Ube3a*^*m*−/*p*+^ mice and WT littermate controls (*Ube3a*^*m+/p+*^) on a congenic C57BL6/J background purchased from The Jackson Laboratory (strain #016590), Bar Harbor, Maine, USA, and bred in a colony in the Children’s National Hospital research core animal facility. Experimenters were blind to genotype during all testing and analysis. Experimental WT and *Ube3a*^*m*−/*p*+^ littermates were generated by crossing female *Ube3a*^*m*+/*p*−^ mice and male WT mice. Two cohorts of mice were used in this study. Cohort 1 included 24 total adult mice aged ~P60–155 (WT male: *n* = 8, *Ube3a*^*m*−/*p*+^ male: *n* = 6, WT female: *n* = 5, *Ube3a*^*m*−/*p*+^ female: *n* = 5). Cohort 2 included 30 total adult mice aged ~P83–114 (WT male: *n* = 8, *Ube3a*^*m*−/*p*+^ male: *n* = 6, WT female: *n* = 9, *Ube3a*^*m*−/*p*+^ female: *n* = 9). We did not exclude any mice from analysis. Ages of mice reported are on day 1 of operant testing.

### Behavioral testing

#### Operant acquisition and extinction.

We used the same testing protocol as was used in our previous study^83^. Mice were food-restricted for the duration of the experiments (2 h of unrestricted feeding after testing each day). We performed daily testing during the light phase at the same time each day. Testing was performed using modular operant conditioning chambers (MED Associates, ENV-307W). These chambers had five available nose-poke apertures on one wall, a food delivery magazine on the opposite wall, and a house light that illuminated the chamber. To provide operant cues, stimulus lights inside the nose-poke apertures were individually controlled. We covered three nose-poke apertures (holes 1, 3, and 5), leaving two open for testing (Fig. 1b). There were three phases of operant testing: magazine training (MAG), operant acquisition (ACQ), and operant extinction (EXT). On the day prior to operant testing, mice were habituated to the chambers with the house light on and no reward delivery for 30 minutes. Next, we trained mice (two 10 min sessions on consecutive days) to receive a food reward (Cohort 1: 20 mg dustless precision pellets, Bio-Serv; Cohort 2: 14 mg dustless precision pellets, Bio-Serv) each time they nose-poked into the food magazine [MAG training]. During operant acquisition (15 min sessions), we illuminated one aperture at a time (the “cued” aperture), and mice received a reward only after a nose-poke into the cued aperture. When a mouse did a nose-poke into the non-illuminated aperture, this was considered a “non-cued” response. For each mouse, the light cue was presented in the same aperture during every trial across days, but cue location (left or right aperture) was randomized across mice. A “trial” is defined as the receipt of a food reward following one or more cued responses, and each trial was self-initiated by the mice, so the number of trials per session was open-ended. To encourage mice to self-initiate more trials, we “primed” mice on by placing one food pellet inside both the cued and non-cued apertures. Priming was performed on the first two days of acquisition and on subsequent sessions following a session with <5 trials. During acquisition, pellet rewards were delivered on a fixed FR1 schedule for the first 10 rewards, then on a variable VR2 schedule for the following rewards. Mice needed to complete >15 trials within a session, and >75% cued response rate [cued responses/(cued + non-cued responses)], over five consecutive days to meet criteria for successful acquisition. Immediately following acquisition, mice underwent three days of extinction training, where the light cue was presented without reward. For analysis of extinction data, we normalized cued and non-cued responses to the group average of the last five days of acquisition. After all mice in a cage completed their last day of operant extinction, mice were returned to normal *ad libitum* food access for the remainder of the study.

#### Ube3a^m−/p+^ behavior battery.

Mice were given 2–4 weeks to recover from operant behavioral testing and food restriction prior to starting the behavior battery. The behavior battery consisted of accelerating rotarod (five consecutive days), open field (one day), marble burying (one day), and nest building (five consecutive days). There was two to three days between the end of one test and the start of the next test. Weight was recorded on the first day of the rotarod prior to behavior testing.

#### Rotarod.

Mice were positioned on a rotating rod that increased speed from 4 to 40 rpm over a five-minute duration with an acceleration of 7.2 rpm^2^pm^2^ (Ugo Basile model #47600). Testing concluded when the animal dropped from the apparatus, completed three successive wrapping rotations, or when the five-minute time limit was reached. Daily testing consisted of two trials separated by a one-hour inter-trial period, with results averaged for each day. The protocol spanned five consecutive days.

#### Open field test.

Mice were introduced into a 42 cm square arena (AccuScan Instruments, Inc., Columbus, OH) and permitted to explore freely during a single 10-minute session. The center zone was 21 cm x 21 cm. Open field activity was monitored using an automatic tracking system (Omnitech Electronics, Inc. SuperFlex Open Field System) employing photocell emitters and receptors that create an x–y grid of infrared beams. Total locomotor distance and duration in the center were measured via infrared beam interruption data. Testing occurred over a single day.

#### Marble burying.

Mice were placed individually in a 16 × 8 in cage with ~4 cm of bedding (Bed-o’Cobs 1/4” bedding) and 20 black glass marbles arranged in a 5 × 4 array for a single 30 min trial. A marble was considered buried if it was >50% covered with bedding at the end of the trial. Testing occurred over a single day. Immediately after marble burying, mice were singly housed for nest building habituation.

#### Nest building.

Immediately after marble burying, mice were then singly-housed and habituated to new nesting material (Bio-Rad 7.5 × 10 cm extra thick block filter paper; 11 – 1 g) for 3 days prior to testing. During testing, new nesting material was introduced on day 1, and unused material was weighed daily across five days.

### Multidimensional analysis of mouse behavior

We used the MATLAB graphical user interface PUMBAA (github.com/sidorovlab/PUMBAA) to perform multidimensional analysis of behavior. Multidimensional analysis using PUMBAA consists of a series of steps: data selection, standardization, principal component analysis (PCA), k-means clustering, and validation, as described in our prior work. Here, we performed multidimensional analysis twice—once with the behavior battery measures alone (Fig. 4a), and a second time with measures from operant behavior also included (Fig. 4g). On the first run, our goal was to determine how accurately PCA would predict genotype if only given data from the *Ube3a*^*m*−/*p*+^ behavior battery. On the second run, we included operant behaviors to determine if these additional behavioral points improved clustering accuracy or the distance between group centroids in PC space. *Data selection*: For the *Ube3a*^*m*−/*p*+^ behavior battery, seven total measures were included: weight, rotarod day 1, rotarod day 5, open field distance traveled, open field center time, marbles buried, and nest building at day 5 (Fig. 4a). For the *Ube3a*^*m*−/*p*+^ behavior battery plus operant extinction (Fig. 4g), 10 total measures were included: *Ube3a*^*m*−/*p*+^ behavior battery (seven measures as above), days to acquisition, accuracy at criteria, and extinction day 1. *Standardization*: All measures were standardized using a z-score (z = (data point − group means)/standard deviation) to account for different units across tests. In prior work, we standardized male and female data separately for tests where a sex difference was observed, using the same method here. *Principal component analysis*: We performed PCA using the pca() function in MATLAB and calculated the amount of variance explained by each PC using a Scree plot and the loading distribution of principal components using the coefficient outputs from PCA (Fig. 4b–c). *k-means clustering*: k-means clustering was performed in principal component space using the kmeans() function in MATLAB with k = 2 clusters. *Validation*: We compared the actual genotypes of the mice to their assigned cluster and calculated the percentage of correct assignments. Centroids of genotype-based groups were calculated by taking the mean of all x and y positions for each mouse in principal component space. Here, centroids were calculated based on the actual genotype, rather than the k-means cluster. The distance between WT and *Ube3a*^*m*−/*p*+^ centroids was defined as the absolute value of the square root of (x_1_-x_2_)^2^ + (y_1_-y_2_)^2^, where x and y represent the coordinates of centroids defined by genotype in 2PC space.

### Statistics

Statistical analysis was performed using GraphPad Prism 9 and MATLAB R2023a (Mathworks). We used unpaired t-tests for the majority of comparisons between WT and *Ube3a*^*m*−/*p*+^ mice when assessing single behavioral timepoints (Figs. 1c, 1e–h). When assessing behavioral outcome measures that occurred over days, we used a two-way RM ANOVA for genotype and day (Figs. 1i, 1k, 1m, 2c, 2f, **S1a**, **S1c**, **S1e**). When assessing the effects of cohort or sex by genotype, we used a two-way ANOVA (Figs. 2a–b, 2d–e, 3a, 3c, 3e–f). When assessing the effects of sex by genotype by days, we used a three-way RM ANOVA (Figs. 3b, 3d). To determine sample sizes needed to achieve a statistically significant result, we performed power analyses of data presented in Figs. 1e, 1h, and 1i (days to acquisition criteria, accuracy at criteria, and E1 extinction). Here, we used MATLAB to conduct a bootstrap simulation using 10,000 iterations per each “n” and set significance to 95% (*α* = 0.05) and power to 80% (1-*β* = 0.8). For all figures, data are represented as mean +/− SEM, were two-tailed, and *p < 0.05, **p < 0.01, ***p < 0.001, and ****p < 0.0001.

## Supplementary Material

Supplement 1

## Figures and Tables

**Table 1.** Bootstrap analysis to guide sample size for operant behavioral testing in *Ube3a* mutants. Sample sizes (“n”) required to detect statistically significant effects (*α* = 0.05) for different levels of power (1-*β* = 0.8, 1-*β* = 0.9, and 1-*β* = 0.95). Behaviors from operant learning and extinction are above the dashed line, and behaviors from the gold standard behavior battery are below the dashed line.

Behavioral measure	Achieved effect size (Cohen’s d)	Sample size per group (*1-β*) *= 0.8*	Sample size per group (*1-β*) *= 0.9*	Sample size per group (*1-β*) *= 0.95*
Days to acquisition	0.84	24	30	>30
Accuracy at criteria	0.89	20	26	>30
Extinction day 1	0.91	21	28	>30

Rotarod day 1	1.64	7	9	11
Rotarod day 5	1.22	12	15	18
Open field distance	0.07	>30	>30	>30
Marbles buried	1.62	7	9	11
Nest building	1.52	9	11	14

## Data Availability

All data generated or analyzed during this study are included in the published article (and its Supplementary Information files).

## References

[1] Bird L. M. Angelman syndrome: review of clinical and molecular aspects. The application of clinical genetics, 93–104 (2014). 24876791 10.2147/TACG.S57386 PMC4036146

[2] Thibert R. L., Larson A. M., Hsieh D. T., Raby A. R. & Thiele E. A. Neurologic manifestations of Angelman syndrome. Pediatr Neurol 48, 271–279 (2013). 10.1016/j.pediatrneurol.2012.09.015 23498559

[3] Sadhwani A. Developmental skills of individuals with Angelman syndrome assessed using the Bayley-III. Journal of autism and developmental disorders 53, 720–737 (2023). 33517526 10.1007/s10803-020-04861-1 PMC8322148

[4] Gwaltney A. Adaptive skills of individuals with angelman syndrome assessed using the Vineland Adaptive Behavior Scales. Journal of Autism and Developmental Disorders 54, 3863–3887 (2024). 37581718 10.1007/s10803-023-06090-8 PMC10867286

[5] Keute M. Angelman syndrome genotypes manifest varying degrees of clinical severity and developmental impairment. Molecular Psychiatry 26, 3625–3633 (2021). 32792659 10.1038/s41380-020-0858-6 PMC8505254

[6] Peters S. U. Cognitive and adaptive behavior profiles of children with Angelman syndrome. American Journal of Medical Genetics Part A 128, 110–113 (2004). 10.1002/ajmg.a.30065 15213998

[7] Willgoss T. Measuring what matters to individuals with Angelman syndrome and their families: Development of a patient-centered disease concept model. Child Psychiatry & Human Development 52, 654–668 (2021). 10.1007/s10578-020-01051-z 32880036 PMC8238699

[8] Kishino T., Lalande M. & Wagstaff J. UBE3A/E6-AP mutations cause Angelman syndrome. Nat Genet 15, 70–73 (1997). 10.1038/ng0197-70 8988171

[9] Clayton-Smith J. & Laan L. Angelman syndrome: a review of the clinical and genetic aspects. J Med Genet 40, 87–95 (2003). 10.1136/jmg.40.2.87 12566516 PMC1735357

[10] Yamasaki K. Neurons but not glial cells show reciprocal imprinting of sense and antisense transcripts of Ube3a. Human molecular genetics 12, 837–847 (2003). 12668607 10.1093/hmg/ddg106

[11] LaSalle J. M., Reiter L. T. & Chamberlain S. J. Epigenetic regulation of UBE3A and roles in human neurodevelopmental disorders. Epigenomics 7, 1213–1228 (2015). 26585570 10.2217/epi.15.70 PMC4709177

[12] Hsiao J. S. et al. A bipartite boundary element restricts UBE3A imprinting to mature neurons. Proceedings of the National Academy of Sciences 116, 2181–2186 (2019). 10.1073/pnas.1815279116 PMC6369781 30674673

[13] Elgersma Y. & Sonzogni M. UBE3A reinstatement as a disease-modifying therapy for Angelman syndrome. Developmental Medicine & Child Neurology 63, 802–807 (2021). 33543479 10.1111/dmcn.14831 PMC8248324

[14] Markati T., Duis J. & Servais L. Therapies in preclinical and clinical development for Angelman syndrome. Expert Opinion on Investigational Drugs 30, 709–720 (2021). 34112038 10.1080/13543784.2021.1939674

[15] Hipp J. F. The UBE3A-ATS antisense oligonucleotide rugonersen in children with Angelman syndrome: a phase 1 trial. Nat Med (2025). 10.1038/s41591-025-03784-7 40646322

[16] Huang H.-S. Topoisomerase inhibitors unsilence the dormant allele of Ube3a in neurons. Nature 481, 185–189 (2012). 10.1038/nature10726 PMC3257422 22190039

[17] Lee H. M. Characterization and structure-activity relationships of indenoisoquinoline-derived topoisomerase I inhibitors in unsilencing the dormant Ube3a gene associated with Angelman syndrome. Mol Autism 9, 45 (2018). 10.1186/s13229-018-0228-2 30140420 PMC6098585

[18] Vihma H. Ube3a unsilencer for the potential treatment of Angelman syndrome. Nature Communications 15, 5558 (2024). 10.1038/s41467-024-49788-8 PMC11231141 38977672

[19] Meng L. Truncation of Ube3a-ATS unsilences paternal Ube3a and ameliorates behavioral defects in the Angelman syndrome mouse model. PLoS genetics 9, e1004039 (2013). 10.1371/journal.pgen.1004039 PMC3873245 24385930

[20] Meng L. Towards a therapy for Angelman syndrome by targeting a long non-coding RNA. Nature 518, 409–412 (2015). 25470045 10.1038/nature13975 PMC4351819

[21] Milazzo C. Antisense oligonucleotide treatment rescues UBE3A expression and multiple phenotypes of an Angelman syndrome mouse model. JCI insight 6 (2021). 10.1172/jci.insight.145991 PMC8410092 34369389

[22] Dindot S. V. An ASO therapy for Angelman syndrome that targets an evolutionarily conserved region at the start of the UBE3A-AS transcript. Science Translational Medicine 15, eabf4077 (2023). 10.1126/scitranslmed.abf4077 36947593

[23] Clarke M. T. Prenatal delivery of a therapeutic antisense oligonucleotide achieves broad biodistribution in the brain and ameliorates Angelman syndrome phenotype in mice. Molecular Therapy 32, 935–951 (2024). 38327047 10.1016/j.ymthe.2024.02.004 PMC11163203

[24] Schmid R. S. CRISPR/Cas9 directed to the Ube3a antisense transcript improves Angelman syndrome phenotype in mice. The Journal of Clinical Investigation 131 (2021). 10.1172/JCI142574 PMC7919720 33411694

[25] Wolter J. M. Cas9 gene therapy for Angelman syndrome traps Ube3a-ATS long non-coding RNA. Nature 587, 281–284 (2020). 33087932 10.1038/s41586-020-2835-2 PMC8020672

[26] Li J. A high-fidelity RNA-targeting Cas13 restores paternal Ube3a expression and improves motor functions in Angelman syndrome mice. Molecular Therapy 31, 2286–2295 (2023). 36805082 10.1016/j.ymthe.2023.02.015 PMC10362381

[27] Daily J. L. Adeno-associated virus-mediated rescue of the cognitive defects in a mouse model for Angelman syndrome. PLoS One 6, e27221 (2011). 10.1371/journal.pone.0027221 PMC3235088 22174738

[28] Judson M. C. Dual-isoform hUBE3A gene transfer improves behavioral and seizure outcomes in Angelman syndrome model mice. JCI insight 6, e144712 (2021). 10.1172/jci.insight.144712 PMC8564914 34676830

[29] Sell G. L. Deleting a UBE3A substrate rescues impaired hippocampal physiology and learning in Angelman syndrome mice. Sci Rep 11, 19414 (2021). 10.1038/s41598-021-97898-w PMC8484563 34593829

[30] Sun A. X. Potassium channel dysfunction in human neuronal models of Angelman syndrome. Science 366, 1486–1492 (2019). 10.1126/science.aav5386 31857479 PMC7735558

[31] Jamal I. Rescue of altered HDAC activity recovers behavioural abnormalities in a mouse model of Angelman syndrome. Neurobiol Dis 105, 99–108 (2017). 10.1016/j.nbd.2017.05.010 28576709

[32] Hu J. H. Activity-dependent degradation of Kv4.2 contributes to synaptic plasticity and behavior in Angelman syndrome model mice. Cell Rep 44, 115583 (2025). 10.1016/j.celrep.2025.115583 PMC12232208 40310720

[33] Sun J. mTORC1-S6K1 inhibition or mTORC2 activation improves hippocampal synaptic plasticity and learning in Angelman syndrome mice. Cell Mol Life Sci 73, 4303–4314 (2016). 10.1007/s00018-016-2269-z 27173058 PMC5056144

[34] Bi X., Sun J., Ji A. X. & Baudry M. Potential therapeutic approaches for Angelman syndrome. Expert Opin Ther Targets 20, 601–613 (2016). 10.1517/14728222.2016.1115837 26558806 PMC4902328

[35] Cao C. Impairment of TrkB-PSD-95 signaling in Angelman syndrome. PLoS Biol 11, e1001478 (2013). 10.1371/journal.pbio.1001478 PMC3570550 23424281

[36] Egawa K. Decreased tonic inhibition in cerebellar granule cells causes motor dysfunction in a mouse model of Angelman syndrome. Sci Transl Med 4, 163ra157 (2012). 10.1126/scitranslmed.3004655 23220633

[37] Pandya N. J. Secreted retrovirus-like GAG-domain-containing protein PEG10 is regulated by UBE3A and is involved in Angelman syndrome pathophysiology. Cell Rep Med 2, 100360 (2021). 10.1016/j.xcrm.2021.100360 PMC8385294 34467244

[38] Cruz E. CIM6P/IGF-2 receptor ligands reverse deficits in Angelman syndrome model mice. Autism Research 14, 29–45 (2021). 33108069 10.1002/aur.2418 PMC8579913

[39] Jiang Y. h. Mutation of the Angelman ubiquitin ligase in mice causes increased cytoplasmic p53 and deficits of contextual learning and long-term potentiation. Neuron 21, 799–811 (1998). 9808466 10.1016/s0896-6273(00)80596-6

[40] Rotaru D. C., Mientjes E. J. & Elgersma Y. Angelman syndrome: from mouse models to therapy. Neuroscience 445, 172–189 (2020). 32088294 10.1016/j.neuroscience.2020.02.017

[41] Bruinsma C. F. Dissociation of locomotor and cerebellar deficits in a murine Angelman syndrome model. J Clin Invest 125, 4305–4315 (2015). 10.1172/JCI83541 26485287 PMC4639977

[42] Heck D. H., Zhao Y., Roy S., LeDoux M. S. & Reiter L. T. Analysis of cerebellar function in Ube3a-deficient mice reveals novel genotype-specific behaviors. Hum Mol Genet 17, 2181–2189 (2008). 10.1093/hmg/ddn117 18413322 PMC2902285

[43] Allensworth M., Saha A., Reiter L. T. & Heck D. H. Normal social seeking behavior, hypoactivity and reduced exploratory range in a mouse model of Angelman syndrome. BMC Genet 12, 7 (2011). 10.1186/1471-2156-12-7 21235769 PMC3025901

[44] Huang H.-S. Behavioral deficits in an Angelman syndrome model: effects of genetic background and age. Behavioural brain research 243, 79–90 (2013). 23295389 10.1016/j.bbr.2012.12.052 PMC3629944

[45] Judson M. C. GABAergic Neuron-Specific Loss of Ube3a Causes Angelman Syndrome-Like EEG Abnormalities and Enhances Seizure Susceptibility. Neuron 90, 56–69 (2016). 10.1016/j.neuron.2016.02.040 27021170 PMC4824651

[46] Gu B. Cannabidiol attenuates seizures and EEG abnormalities in Angelman syndrome model mice. J Clin Invest 129, 5462–5467 (2019). 10.1172/JCI130419 31503547 PMC6877312

[47] Mandel-Brehm C., Salogiannis J., Dhamne S. C., Rotenberg A. & Greenberg M. E. Seizure-like activity in a juvenile Angelman syndrome mouse model is attenuated by reducing Arc expression. Proc Natl Acad Sci U S A 112, 5129–5134 (2015). 10.1073/pnas.1504809112 25848016 PMC4413330

[48] Shi S. Q., Bichell T. J., Ihrie R. A. & Johnson C. H. Ube3a imprinting impairs circadian robustness in Angelman syndrome models. Curr Biol 25, 537–545 (2015). 10.1016/j.cub.2014.12.047 25660546 PMC4348236

[49] Ehlen J. C. Maternal Ube3a Loss Disrupts Sleep Homeostasis But Leaves Circadian Rhythmicity Largely Intact. J Neurosci 35, 13587–13598 (2015). 10.1523/JNEUROSCI.2194-15.2015 26446213 PMC4595617

[50] Copping N. A. & Silverman J. L. Abnormal electrophysiological phenotypes and sleep deficits in a mouse model of Angelman Syndrome. Mol Autism 12, 9 (2021). 10.1186/s13229-021-00416-y 33549123 PMC7866697

[51] Colas D., Wagstaff J., Fort P., Salvert D. & Sarda N. Sleep disturbances in Ube3a maternal-deficient mice modeling Angelman syndrome. Neurobiol Dis 20, 471–478 (2005). 10.1016/j.nbd.2005.04.003 15921919

[52] Sidorov M. S. Delta rhythmicity is a reliable EEG biomarker in Angelman syndrome: a parallel mouse and human analysis. J Neurodev Disord 9, 17 (2017). 10.1186/s11689-017-91958 28503211 PMC5422949

[53] Spencer E. R. Longitudinal EEG model detects antisense oligonucleotide treatment effect and increased UBE3A in Angelman syndrome. Brain Commun 4, fcac106 (2022). 10.1093/braincomms/fcac106 PMC9123847 35611307

[54] Born H. A. Strain-dependence of the Angelman Syndrome phenotypes in Ube3a maternal deficiency mice. Scientific reports 7, 8451 (2017). 28814801 10.1038/s41598-017-08825-x PMC5559514

[55] Montgomery D. P., Shide J. J., Didouchevski A. M., Dickinson A. H. & Sidorov M. S. Periodic and aperiodic contributions to EEG delta power are translatable and complementary Angelman syndrome biomarkers. bioRxiv (2025). 10.1101/2025.09.08.674941 PMC13661899 42321460

[56] Perrino P. A., Chamberlain S. J., Eigsti I. M. & Fitch R. H. Communication-related assessments in an Angelman syndrome mouse model. Brain and Behavior 11, e01937 (2021). 10.1002/brb3.1937 PMC7821623 33151040

[57] Guoynes C. D., Pavalko G. & Sidorov M. S. Courtship and distress ultrasonic vocalizations are altered in a mouse model of Angelman syndrome. J Neurodev Disord 17, 59 (2025). 10.1186/s11689-025-09648-y 41034775 PMC12486785

[58] Sonzogni M. A behavioral test battery for mouse models of Angelman syndrome: a powerful tool for testing drugs and novel Ube3a mutants. Molecular autism 9, 1–19 (2018). 30220990 10.1186/s13229-018-0231-7 PMC6137919

[59] Qu S. Dietary Intake of Octanoic Acid Restores UBE3A Expression and Improves the Behavioral Phenotypes in a Mouse Model of Angelman Syndrome. The FASEB Journal 39, e70559 (2025). 10.1096/fj.202403130RR PMC12023718 40277210

[60] Silva-Santos S. Ube3a reinstatement identifies distinct developmental windows in a murine Angelman syndrome model. J Clin Invest 125, 2069–2076 (2015). 10.1172/JCI80554 25866966 PMC4463212

[61] Sonzogni M., Zhai P., Mientjes E. J., van Woerden G. M. & Elgersma Y. Assessing the requirements of prenatal UBE3A expression for rescue of behavioral phenotypes in a mouse model for Angelman syndrome. Mol Autism 11, 70 (2020). 10.1186/s13229-020-00376-9 32948244 PMC7501605

[62] Sonzogni M. Delayed loss of UBE3A reduces the expression of Angelman syndrome-associated phenotypes. Mol Autism 10, 23 (2019). 10.1186/s13229-019-0277-1 31143434 PMC6532248

[63] Dodge A. Generation of a novel rat model of Angelman syndrome with a complete Ube3a gene deletion. Autism Research 13, 397–409 (2020). 31961493 10.1002/aur.2267 PMC7787396

[64] Berg E. Translational outcomes in a full gene deletion of ubiquitin protein ligase E3A rat model of Angelman syndrome. Translational psychiatry 10, 39 (2020). 32066685 10.1038/s41398-020-0720-2 PMC7026078

[65] Myers L. S. A preclinical pig model of Angelman syndrome mirrors the early developmental trajectory of the human condition. Proceedings of the National Academy of Sciences 122, e2505152122 (2025). 10.1073/pnas.2505152122 PMC12318228 40690672

[66] Moreira-de-Sá A. Adenosine A2A receptors format long-term depression and memory strategies in a mouse model of Angelman syndrome. Neurobiology of Disease 146, 105137 (2020). 10.1016/j.nbd.2020.105137 33049319

[67] Schultz M. N. & Crawley J. N. Evaluation of a TrkB agonist on spatial and motor learning in the Ube3a mouse model of Angelman syndrome. Learning & Memory 27, 346–354 (2020). 32817301 10.1101/lm.051201.119 PMC7433657

[68] Guzzetti S. Taurine administration recovers motor and learning deficits in an Angelman syndrome mouse model. International journal of molecular sciences 19, 1088 (2018). 29621152 10.3390/ijms19041088 PMC5979575

[69] Berg E. L. Insulin-like growth factor-2 does not improve behavioral deficits in mouse and rat models of Angelman syndrome. Molecular Autism 12, 1–16 (2021). 34526125 10.1186/s13229-021-00467-1 PMC8444390

[70] Liu Y. Enhancement of synaptic plasticity and reversal of impairments in motor and cognitive functions in a mouse model of Angelman Syndrome by a small neurogenic molecule, NSI-189. Neuropharmacology 144, 337–344 (2019). 30408487 10.1016/j.neuropharm.2018.10.038

[71] Kaphzan H. Reversal of impaired hippocampal long-term potentiation and contextual fear memory deficits in Angelman syndrome model mice by ErbB inhibitors. Biological psychiatry 72, 182–190 (2012). 22381732 10.1016/j.biopsych.2012.01.021 PMC3368039

[72] Vorhees C. V. & Williams M. T. Morris water maze: procedures for assessing spatial and related forms of learning and memory. Nature protocols 1, 848–858 (2006). 17406317 10.1038/nprot.2006.116 PMC2895266

[73] Phillips R. & LeDoux J. E. Differential contribution of amygdala and hippocampus to cued and contextual fear conditioning. Behavioral neuroscience 106, 274 (1992). 1590953 10.1037//0735-7044.106.2.274

[74] Broadbent N. J., Gaskin S., Squire L. R. & Clark R. E. Object recognition memory and the rodent hippocampus. Learning & memory 17, 5–11 (2010). 20028732 10.1101/lm.1650110 PMC2807177

[75] Koike H. Chemogenetic inactivation of dorsal anterior cingulate cortex neurons disrupts attentional behavior in mouse. Neuropsychopharmacology 41, 1014–1023 (2016). 26224620 10.1038/npp.2015.229 PMC4748426

[76] Norman K. J. Chemogenetic suppression of anterior cingulate cortical neurons projecting to the visual cortex disrupts attentional behavior in mice. Neuropsychopharmacology Reports 41, 207–214 (2021). 33955711 10.1002/npr2.12176 PMC8340833

[77] Green J. T. & Bouton M. E. New functions of the rodent prelimbic and infralimbic cortex in instrumental behavior. Neurobiology of learning and memory 185, 107533 (2021). 10.1016/j.nlm.2021.107533 PMC8653515 34673264

[78] Miller E. K. & Cohen J. D. An integrative theory of prefrontal cortex function. Annu Rev Neurosci 24, 167–202 (2001). 10.1146/annurev.neuro.24.1.167 11283309

[79] Le Merre P., Ahrlund-Richter S. & Carlen M. The mouse prefrontal cortex: Unity in diversity. Neuron 109, 1925–1944 (2021). 10.1016/j.neuron.2021.03.035 33894133

[80] Rotaru D. C., van Woerden G. M., Wallaard I. & Elgersma Y. Adult Ube3a Gene Reinstatement Restores the Electrophysiological Deficits of Prefrontal Cortex Layer 5 Neurons in a Mouse Model of Angelman Syndrome. J Neurosci 38, 8011–8030 (2018). 10.1523/JNEUROSCI.0083-18.2018 30082419 PMC6596147

[81] Sidorov M. S. Visual Sequences Drive Experience-Dependent Plasticity in Mouse Anterior Cingulate Cortex. Cell Rep 32, 108152 (2020). 10.1016/j.celrep.2020.108152 PMC7536640 32937128

[82] Negron-Moreno P. N., Diep D. T., Guoynes C. D. & Sidorov M. S. Dissociating motor impairment from five-choice serial reaction time task performance in a mouse model of Angelman syndrome. Front Behav Neurosci 16, 968159 (2022). 10.3389/fnbeh.2022.968159 PMC9539753 36212189

[83] Sidorov M. S. Enhanced operant extinction and prefrontal excitability in a mouse model of Angelman syndrome. Journal of Neuroscience 38, 2671–2682 (2018). 29431654 10.1523/JNEUROSCI.2828-17.2018 PMC5852653

[84] Tanas J. K. Multidimensional analysis of behavior predicts genotype with high accuracy in a mouse model of Angelman syndrome. Translational psychiatry 12, 426 (2022). 36192373 10.1038/s41398-022-02206-3 PMC9529912

[85] Koyavski L. Sex-dependent sensory phenotypes and related transcriptomic expression profiles are differentially affected by Angelman syndrome. Molecular Neurobiology 56, 5998–6016 (2019). 30706369 10.1007/s12035-019-1503-8

[86] Cosgrove J. A., Kelly L. K., Kiffmeyer E. A. & Kloth A. D. Sex-dependent influence of postweaning environmental enrichment in Angelman syndrome model mice. Brain and Behavior 12, e2468 (2022). 10.1002/brb3.2468 PMC8865162 34985196

[87] Jamal I. Environmental enrichment improves behavioral abnormalities in a mouse model of Angelman syndrome. Molecular neurobiology 54, 5319–5326 (2017). 27581300 10.1007/s12035-016-0080-3

[88] Finger E. C., Mitchell D. G., Jones M. & Blair R. J. R. Dissociable roles of medial orbitofrontal cortex in human operant extinction learning. Neuroimage 43, 748–755 (2008). 18793731 10.1016/j.neuroimage.2008.08.021 PMC3941699

[89] Alderman N. Central executive deficit and response to operant conditioning methods. Neuropsychological Rehabilitation 6, 161–186 (1996).

[90] Krueger D. D., Osterweil E. K., Chen S. P., Tye L. D. & Bear M. F. Cognitive dysfunction and prefrontal synaptic abnormalities in a mouse model of fragile X syndrome. Proceedings of the National Academy of Sciences 108, 2587–2592 (2011). 10.1073/pnas.1013855108 PMC3038768 21262808

[91] Guoynes C. D., Pavalko G. & Sidorov M. S. Courtship and distress ultrasonic vocalizations are disrupted in a mouse model of Angelman syndrome. Research Square, rs. 3. rs-5953744 (2025). 10.1186/s11689-025-09648-y PMC12486785 41034775

[92] Bolivar V. J., Caldarone B. J., Reilly A. A. & Flaherty L. Habituation of activity in an open field: a survey of inbred strains and F1 hybrids. Behavior genetics 30, 285–293 (2000). 11206083 10.1023/a:1026545316455

[93] Rudeck J., Vogl S., Banneke S., Sch nfelder G. & Lewejohann L. Repeatability analysis improves the reliability of behavioral data. PloS one 15, e0230900 (2020). 10.1371/journal.pone.0230900 PMC7117744 32240211

[94] Kilkenny C., Browne W., Cuthill I. C., Emerson M. & Altman D. G. Animal research: reporting in vivo experiments: the ARRIVE guidelines. British journal of pharmacology 160, 1577 (2010). 20649561 10.1111/j.1476-5381.2010.00872.x PMC2936830

