# Supplementary material for: Operant behavior is reliably impaired in a mouse model of Angelman syndrome
Source: Res Sq. 2026 Mar 20:rs.3.rs-9043922. Preprint. [Version 1] doi: 10.21203/rs.3.rs-9043922/v1 (PMC13015589; doi:10.21203/rs.3.rs-9043922/v1)
Supplement: Supplement 1 [file NIHPPrs9043922v1-supplement-1.pdf]

Supplementary Figure S1. Raw data during extinction. Data are associated with extinction reported as normalized to baseline in Figure 1. Black: WT, red: *Ube3a*$^{m-/p+}$ (AS). (a) Cued responses during acquisition and extinction. *Ube3a*$^{m-/p+}$ mice had fewer cued responses during the five days at criteria in the acquisition phase, represented as an average of five days in “ACQ” (as reported in Fig. 1g). During the extinction phase, analysis of raw responses revealed a main effect of genotype ($F_{(1,54)} = 25.00$, $p < 0.0001$) and a day × genotype interaction ($F_{(2,108)} = 9.292$, $p = 0.0002$). Bracket represents interaction and asterisks represent *post hoc* tests (E1 $p < 0.0001$, E2 $p = 0.1781$, E3 $p = 0.0117$). (b) Cued responses during extinction in individual mice. (c) Non-cued responses during acquisition and extinction. *Ube3a*$^{m-/p+}$ had fewer non-cued responses during the five days at criteria in the acquisition phase, represented as an average of five days in “ACQ” (as reported in Fig. 1g). During the extinction phase, analysis of raw responses revealed a main effect of genotype ($F_{(1,58)} = 20.66$, $p < 0.0001$) but no day × genotype interaction ($F_{(2,116)} = 0.3888$, $p = 0.6788$). Asterisks represent *post hoc* tests (E1 $p = 0.0021$, E2 $p = 0.0091$, E3 $p = 0.0316$). (d) Non-cued responses during extinction in individual mice. (e) Accuracy [cued responses / (cued responses + non-cued responses)] during acquisition and extinction. *Ube3a*$^{m-/p+}$ mice had higher accuracy during the five days at criteria in the acquisition phase, represented as an average of five days in “ACQ” (as reported in Fig. 1h). During the extinction phase, analysis of accuracy revealed a main effect of genotype ($F_{(1,54)} = 9.046$, $p = 0.0040$) but no day × genotype interaction ($F_{(2,108)} = 1.128$, $p = 0.3276$). Asterisks represent *post hoc* tests (E1 $p = 0.0356$, E2 $p = 0.0247$, E3 $p = 0.1677$). (f) Accuracy during extinction in individual mice. Data represent mean ± SEM; ****$p < 0.0001$; ***$p < 0.001$; **$p < 0.01$; *$p < 0.05$.

Supplementary File S1. Statistics for all figures. Data represent mean ± SEM; ****$p < 0.0001$; ***$p < 0.001$; **$p < 0.01$; *$p < 0.05$.
